# BCREval: a computational method to estimate the bisulfite conversion ratio in WGBS

**DOI:** 10.1186/s12859-019-3334-z

**Published:** 2020-01-31

**Authors:** Junhua Zhou, Minqiong Zhao, Zefang Sun, Feilong Wu, Yucong Liu, Xianghua Liu, Zuping He, Quanze He, Quanyuan He

**Affiliations:** 1 School of Medicine, Hunan Normal University, Tongzipo Road 371, Changsha, 410013 People’s Republic of China; 2 Verna and Marrs McLean Department of Biochemistry and Molecular Biology, Baylor College of Medicine One Baylor Plaza, Houston, TX 77-30 USA; 3 The Affiliated Suzhou Hospital of Nanjing Medical University, Suzhou, People’s Republic of China

**Keywords:** DNA methylation, Whole genome bisulfite sequencing (WGBS), Bisulfite conversion ratio (BCR), Telomere

## Abstract

**Background:**

Whole genome bisulfite sequencing (WGBS) also known as BS-seq has been widely used to measure the methylation of whole genome at single-base resolution. One of the key steps in the assay is converting unmethylated cytosines into thymines (BS conversion). Incomplete conversion of unmethylated cytosines can introduce false positive methylation call. Developing a quick method to evaluate bisulfite conversion ratio (BCR) is benefit for both quality control and data analysis of WGBS.

**Results:**

Here we provide a computational method named “BCREval” to estimate the unconverted rate (UCR) by using telomeric repetitive DNA as native spike-in control. We tested the method by using public WGBS data and found that it is very stable and most of BS conversion assays can achieve> 99.5% efficiency. The non-CpG DNA methylation at telomere fits a binomial model and may result from a random process with very low possibility (the ratio < 0.4%). And the comparison between BCREval and Bismark (Krueger and Andrews, Bioinformatics 27:1571–1572, 2011), a widely used BCR evaluator, suggests that our algorithm is much faster and more efficient than the latter.

**Conclusion:**

Our method is a simple but robust method to QC and speculates BCR for WGBS experiments to make sure it achieves acceptable level. It is faster and more efficient than current tools and can be easily integrated into presented WGBS pipelines.

## Background

DNA methylation, as a fundamental epigenetics modification, plays critical roles in various biology processes including embryonic development, pluripotency maintenance, genomic imprinting, gene expression regulation, and genomic stability maintenance. It involves the addition of a methyl group to the carbon 5 position of CpG (most of tissues) and non-CpG dinucleotides (embryonic stem cell et.al.) by DNA methyltransferases in a tissue-specific way. Knowledge of the genomic methylation landscape is essential for understanding how methylation patterns are established and maintained and the significance of DNA methylation in development and disease.

Several methods exist for measuring DNA methylation in genomic wide including Whole genome bisulfite sequencing (WGBS), reduced representation bisulfite sequencing (RRBS), post-bisulfite adapter tagging (PBAT) [1] and Methylated DNA immunoprecipitation based methods (MeDIP-chip and MeDIP-seq) [2]. In which, WGBS is the only one to identify all the C information and global pattern therefore has become the standard profiling method in major epigenome consortiums ﻿such as NIH Roadmap [3], ENCODE [4], Blueprint [5] and IHEC [6]. In this assay, genomic DNA is purified and sheared into fragments and then treated with bisulfite, a chemical that converts unmethylated cytosine but not methylated ones to uracil. The bisulfite converted genomic DNA is then sequenced by a Next-generation sequencing platform. The methylation states of cytosines are determined by searching T-C mismatches between sequences obtained and the reference genome.

In a WGBS assay, it was implicitly assumed that this bisulfite conversion should run to completion. However, it is not always the case. Incomplete conversion of unmethylated cytosines will make it impossible to distinguish unconverted unmethylated cytosines from methylated ones and therefore will result in false positive methylation calls^7^. At other hand, prolonged bisulfite treatment causes DNA degradation in a way of remaining methylated reads. So it is important to estimate bisulfite conversion ratio (BCR) for each WGBS experiment.

Some studies have used observed BCR in closed non-CpG sites to estimate BCR by assuming very low methylation ratio of non-CpG sites which may not be the case especially in some cell type (e.g. ES cell). Additionally, some (C/T) SNPs and low coverage regions may also introduce noise for the strategy. Another option to estimate BCR is using spike-in control of nonnative DNA with a known methylation state, which increases the complexity of procedure and rely on another assumption that DNA and spike DNA have the same conversion properties which is also questionable.

Telomeres are distinctive structures found at the ends of chromosomes, which protect the ends of chromosomes from deterioration or fusion with neighboring chromosomes. In vertebrate telomeres, the sequence of TTAGGG is repeated strictly approximate 3000 times and can reach up to 15,000 base pairs in length. Its complementary DNA strand contains CCCTAA repeats which have three non-CpG sites (one CpT and two CpC sites) for each repeat. As there are a lot of non-CpG site exist in telomeres, using telomeric DNA as native spike-in control may be a better way to evaluate BCR.

Here, we provide a computational method named “BCREval” to evaluate BCR using telomeric DNA as native spike-in control for WGBS experiment. We tested the method using 11 public available WGBS data of various cell lines and tissues and found that this method is easy to use and stable. In most of cases, the BCR of telomeric DNA is above 99.5% suggesting that telomeric DNA are unmethylated in all test samples and the DNA methylation at telomere fits a binomial model and may result from a random process with very low possibility. Interestedly, no significant differences of non-CpG methylation ratio was found in three non-CpG cytosine sites. Additionally, the comparison between BCREval and Bismark [8], a widely used BCR evaluator, shows that our algorithm is much faster and more efficient than the latter. Finally, the python script implementing the method is ready and easy to integrate into presented WGBS pipelines.

## Results

**Fig. 1:**
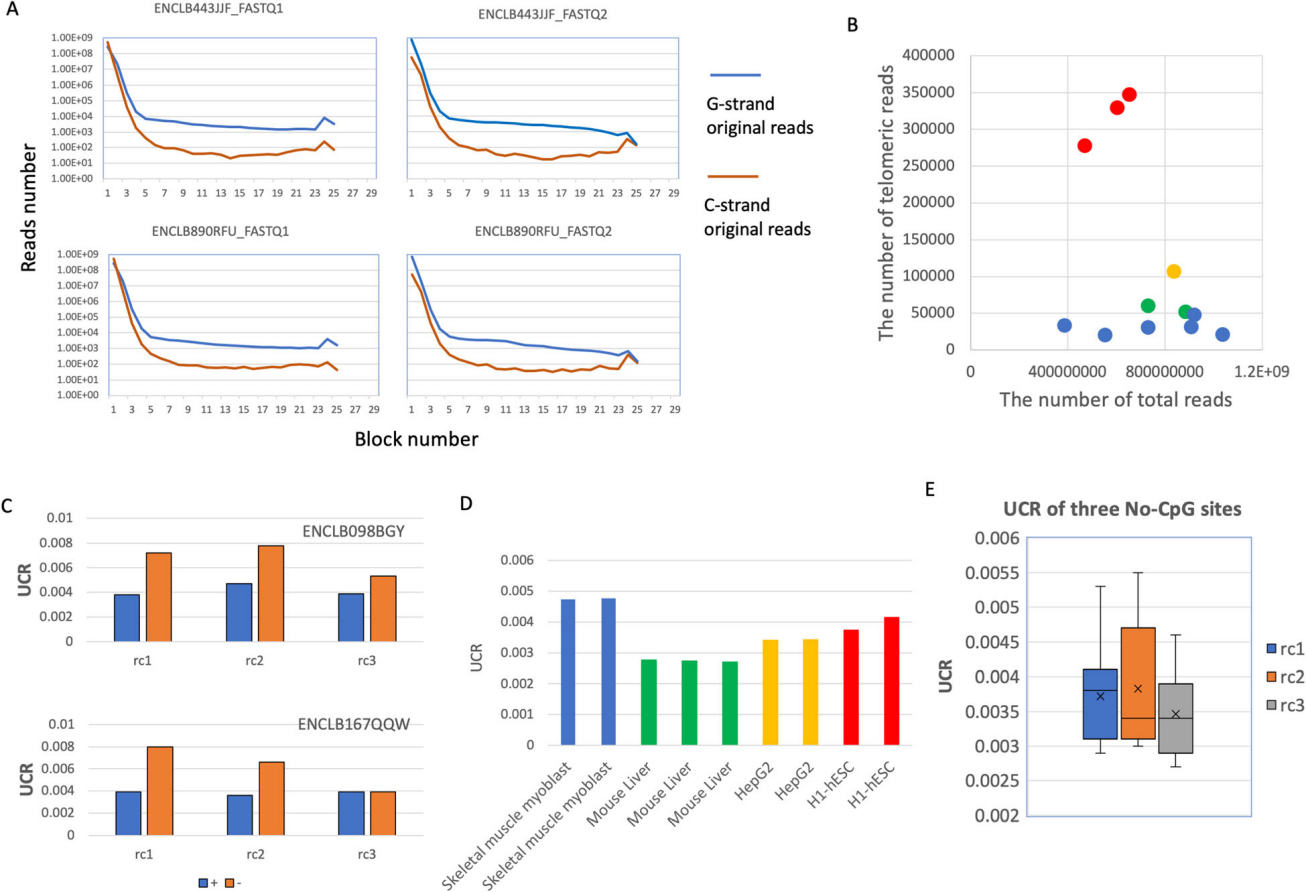
The characteristics of bisulfite converted telomeric WGSBS data. **a** The distribution of read with distinct number of telomeric blocks in two sample (ENCLB443JJF and ENCLB890RFU). The G-strand and C-strand original reads are color blue and orange respectively. FASTQ1 and FASTQ2 indicate the file containing forward and reversed reads in paired-end NGS sequencing; **b** A scatter plot showing the numbers of telomeric reads and total reads of 12 WGSBS experiments (red and orange dots represent tissue samples); **c** UCRs calculated by using forward (+) and reversed (-) reads in three cytosines sites in two samples (ENCLB098BGY, ENCLB167QQW); **d** The UCRs of technical repeats of two tissues (skeletal muscle myoblast and mouse liver) and two cell lines (HepG2 and H1-hESC); **e** The box-plot showing the distribution of UCR of three cytosines sites across eleven samples.

We counted the number of reads with n repeated blocks (n range from 1 to 30) in both forward and reversed FASTQ files (Fig. 1a). As expected, along with the increasing of n, the number of matched reads decreases dramatically and then becomes stable after *n* > =8 (Fig. 1a). Therefore we used 8 as the minimal repeated block number to distinguish telomeric reads from others. It is consistence with the fact that 58 of 59 (TTAGGG) locate on either subtelomeric or telomeric regions in human genome (hg38) (Additional file 1: Figure S1). The peaks at *n* = 24~25 indicates that many reads are composed by telomeric repeats completely and suggests the ability of WGBS to capture telomeric DNA. Interestedly, the data also show that the G strand original telomeric reads (50% GC contents) are much more than ones from C strand (low GC contents) in both FASTQ files, which may result in the GC bias of PCR step (Fig. 1a). Interestedly, comparing with cell-lines, all tissue samples have higher ratios of telomeric reads to total ones (Fig. 1b).

The unconverted ratio (UCR) of three non-CpG sites of telomeric reads were calculated for both forward and reversed FASTQ files. We found that UCRs from reversed FASTQs file frequently slightly higher than their count parts from forward FASTQs (Fig. 1c). The possible reason is that all reversed telomeric reads were always sequenced from telomeres to centromeres which may introduce false telomeric blocks at the end of reads. Therefore, we only used forward FASTQ files for further analyses. We also found that the variants of UCRs among technical replicates are very small suggesting good reliability of the method (Fig. 1d).

**Fig. 2:**
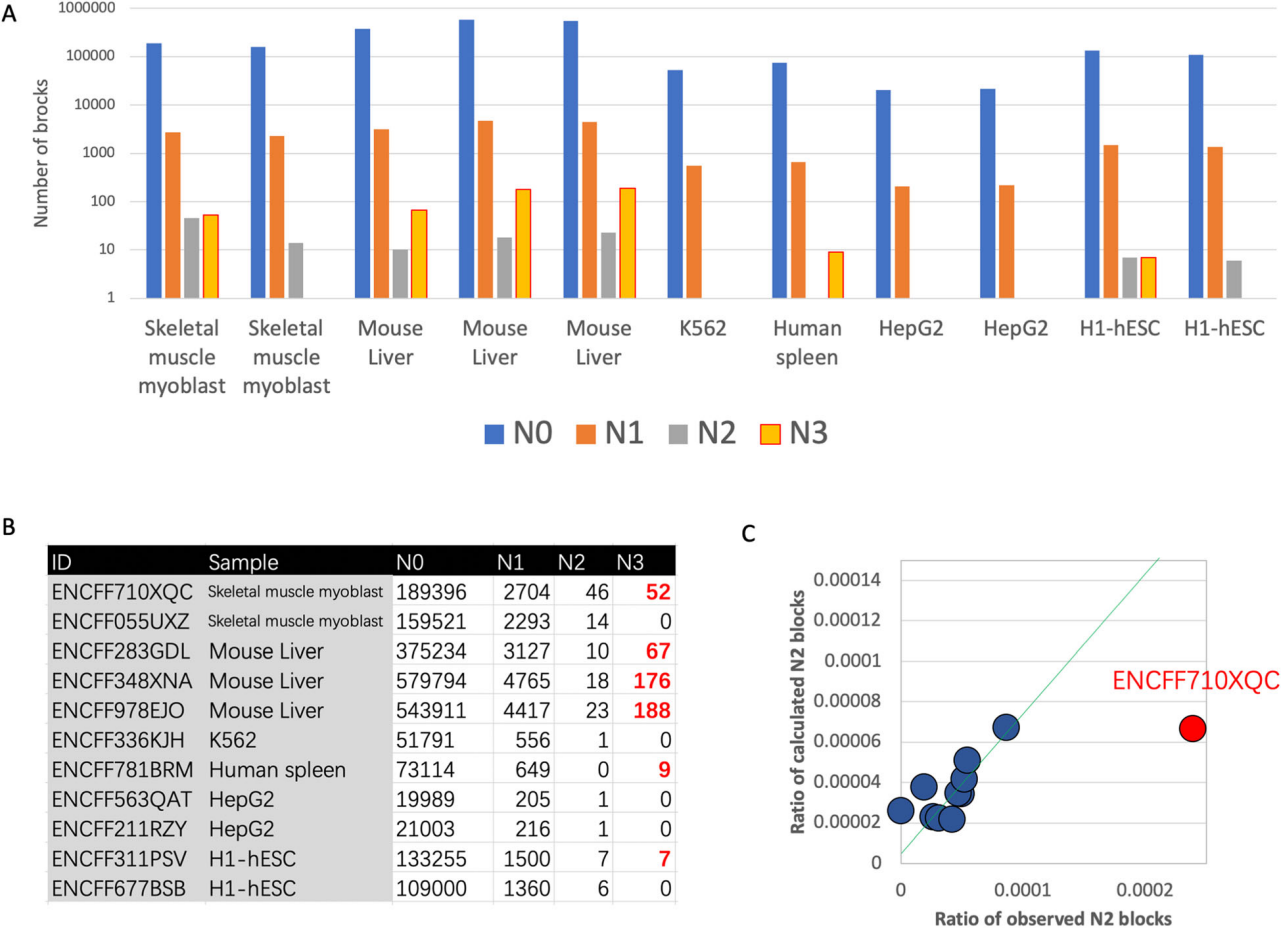
The distribution of telomeric repeat blocks. **a** Bar chart showing the distribution of telomeric repeat block (N0~N3 blocks) in eleven samples. Note, the y axis was log10 transformed; **b** The raw data of the figure 4A, in which the numbers of N3 block above zero are highlighted as red; **c** The dot plot of the calculated ratio of N2 blocks against observed ones. Each dot represents a sample and the green trend line was calculated only using blue dots.

Methylated cytosines are found primarily at CpG dinucleotides, but are also found at non-CpG sites (CpA, CpT and CpC) in specific mammal cell types including pluripotent stem cells, oocytes, neurons, and glial cells [7]. Using our method, we found that in most cases, the unconverted cytosines in telomere are rare (UCR < 0.5%) and no significant difference of UCRs were found among three non-CpG sites (Fig. 1e), suggesting the bisulfite conversion treatment is very realizable and has high efficiency (> 99.5%). However, we do observe that 1.1%~ 1.7% telomeric blocks have unconverted cytosines in all samples, which may result from either the failure of bisulfite conversion or cytosine methylation. The UCRs of telomere blocks were solved using the formula 2 and then R2s and R3s were calculated using formula 3 and 4 respectively. As the data shown, with the exception of ENCFF710XQC, there is a good positive correlation between observed and calculated R2s(Fig. 2a,b) and their paired-values are comparable (Fig. 2c), which suggests that the binomial model fits these data well and the cytosine methylation are non-specific and random events in these samples.

Based on the random model, the expected number of R3 blocks should be zero for all samples (Additional file 1: Table S1). However, there are six of eleven samples have N3 blocks, which may result from either untreated genomic DNA contamination or enzymatic methylation. It is easy to distinguish them using distribution patterns, because the N3 blocks from contamination usually cluster together and enzymatic methylated ones should be dispersed. As the data shown that all N3 blocks from four samples (ENCFF710XQC, ENCFF283GDL, ENCFF348XNA, ENCFF978EJO) cluster into several N3 block richen reads suggesting untreated genomic DNA contamination, which may result in false methylation calls. As N3 blocks were observed in ENCFF710XQC but not its technical repeat ENCFF055UXZ, it also suggests that these 52 N3 blocks may not be relevant to biology but result from technical noise (Fig. 2b).

To evaluate the performance of “BCREval”, we compared it with the existing BCR evaluator Bismark [8]. The results show that the methylation levels of CpCs in genome-wide (about 0.8%) are in general higher than that in telomeres (< 0.65%). And our algorithm has less recourse consumption (only 44 M Memory Usage), higher speed (30x faster) and comparable accuracy if not better (Table 1), which suggests the advantages of BCREval to be integrated into current WGBS pipelines. The major reason is that Bismark elucidates non-CpG methylation levels by aligning all reads to a bisulfite converted genome, which depends on aligner (for example Bowtie2) and is time/resource consuming.

**Table 1:** The performance comparison of BCREval and Bismark

ENCODE ID	File Size	Reads Number	Processing Time		Memory Usage		CHH methylation ratio %	
			Bismark	BCREval	Bismark	BCREval	Bismark	BCREval
ENCFF055UXZ	1.1G	12 M	4 h 49 m	10 m	10G	44 M	0.7	0.56
ENCFF336KJH	687 M	12 M	4 h 8 m	9 m	10G	44 M	0.5	0.54
ENCFF677BSB	926 M	12 M	5 h 28 m	9 m	10G	44 M	1.1	0.42
ENCFF781BRM	833 M	12 M	5 h 5 m	9 m	10G	44 M	0.5	0.26
ENCFF710XQC	1011 M	12 M	5 h 5 m	10 m	10G	44 M	0.8	0.45
ENCFF211RZY	1.1G	12 M	4 h 39 m	10 m	10G	44 M	0.5	0.17
ENCFF563QAT	821 M	12 M	4 h 31 m	8 m	10G	44 M	0.5	0.18
ENCFF311PSV	686 M	12 M	4 h 36 m	10 m	10G	44 M	1.1	0.3

## Discussion

In a WGBS experiment, researchers implicitly assume that all unmethylated cytosines are converted into thymines, which unfortunately may not be the case. Based on our knowledge current methods to monitor bisulfite conversion rate in WGBS experiments are time/resource consuming and may introduce false methylation calls. Here, we present a simple but robust method to QC and speculate BCR for WGBS experiments by using non-CpG sites in telomeric DNA as natural/internal controls.

We speculated 11 samples using our method and found: 1) Bisulfite conversion assay is really stable and usually achieves > 99.5% efficiency; 2) In most cases, the cytosine methylation at telomeres is a random processes and the methylation rates of telomere usually are very small (< 0.4%); 3) No locational or sequence preferences are found in the three non-CpG sites; 4) Although it is impossible to distinguish random cytosine methylation from failure of bisulfite conversion in WGBS, our method have the potential to detect enzymatic DNA methylation by comparing the distribution of N1 and N2 blocks; 5) Our method has the ability to detect trace untreated genomic DNA contamination for QC purpose.

More importantly, comparing with existing BCR evaluator Bismark [8], BCREval has many advantages including faster processing, less recourse consumption and is easier integrated into presented WGBS pipelines. Although it should be kept in mind that ﻿telomeres, as native/inner control, might not necessarily have the same conversion properties and base non-CpG site methylation level as other genomic DNA, telomeric ﻿methylation state may still be a good indicator of baseline of no-CpG methylation and global DNA methylation dynamics.

Additionally, because all mammals share the same telomere DNA sequence, this method should be applicable to nonhuman mammalian samples without any modifications. it is also easy to modify program for other species with distinct telomere repeats by following the same principle of our method.

## Conclusion

BCREval is a simple but robust method to speculates BCR for WGBS experiments to make sure it achieves acceptable level. It is much faster and more efficient than existing tools and can be easily integrated into current WGBS pipelines. A python script to implement BCREval is freely available at https://github.com/hqyone/BCR_Evaluator.

## Methods

All WGBS testing data (FASTQ files) were download form ENCODE database including 2 cell lines, 1 primary cell and 3 tissues (Table 2), All of them are pair-end WGBS data and have at least 20 M reads with length above 100 bp. The home-made python script which implemented our algorithm can be download from here and has the ability to process multiple FASTQ files in a batch way. Detailed results can be found in a text output file.

**Table 2:** The data using in the manuscript

Biosample Type	Library_ID	ENCODE_ID (FASTQ)	Strand	Biosample summary
Primary cell	ENCLB587BLQ	ENCFF055UXZ	**+**	*Homo sapiens* skeletal muscle myoblast
	ENCLB587BLQ	ENCFF764NTF	**–**	*Homo sapiens* skeletal muscle myoblast
	ENCLB988SSO	ENCFF710XQC	**+**	*Homo sapiens* skeletal muscle myoblast
	ENCLB988SSO	ENCFF331AID	**–**	*Homo sapiens* skeletal muscle myoblast
Cell line	ENCLB542OXH	ENCFF336KJH	**+**	*Homo sapiens* K562
	ENCLB542OXH	ENCFF585HYM	**–**	*Homo sapiens* K562
	ENCLB890RFU	ENCFF211RZY	**+**	*Homo sapiens* HepG2
	ENCLB890RFU	ENCFF717MDZ	**–**	*Homo sapiens* HepG2
	ENCLB443JJF	ENCFF563QAT	**+**	*Homo sapiens* HepG2
	ENCLB443JJF	ENCFF954LFD	**–**	*Homo sapiens* HepG2
Stem cell	ENCLB098BGY	ENCFF677BSB	**+**	*Homo sapiens* H1-hESC
	ENCLB098BGY	ENCFF800KIP	**–**	*Homo sapiens* H1-hESC
	ENCLB167QQW	ENCFF311PSV	**+**	*Homo sapiens* H1-hESC
	ENCLB167QQW	ENCFF335TUD	**–**	*Homo sapiens* H1-hESC
Tissue	ENCLB353RJB	ENCFF781BRM	**+**	*Homo sapiens* spleen male adult (37 years)
	ENCLB353RJB	ENCFF535VCB	**–**	*Homo sapiens* spleen male adult (37 years)
	ENCLB585SDT	ENCFF283GDL	**+**	*Mus musculus* C57BL/6 liver adult (54–61 day)
	ENCLB506AYR	ENCFF978EJO	**+**	*Mus musculus* C57BL/6 liver adult (54–61 day)
	ENCLB760KHX	ENCFF348XNA	**+**	*Mus musculus* C57BL/6 liver adult (54–61 day)

**Fig. 3:**
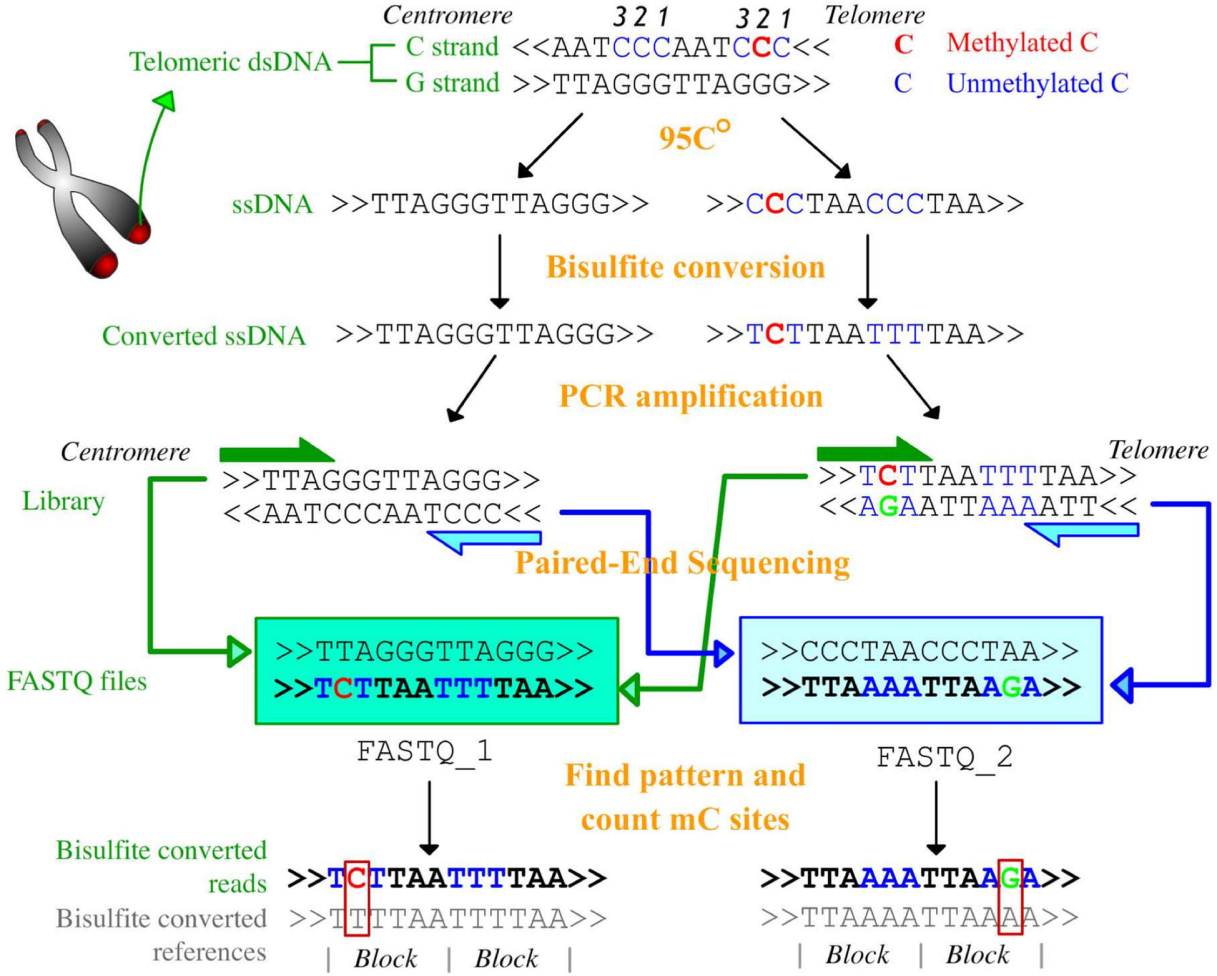
The procedure of telomeric DNA bisulfite conversion and paired-end sequencing. The treatments and their productions are labeled as orange and green. The methylated and unmethylated cytosines in sequences are colored as red and blue respectively. The labels “centromere” and “telomere” indicate the direction of sequences or reads in genome. The numbers above C indicate the indexes of three non-CpG cytosines in telomeric DNA blocks.

**Fig. 4:**
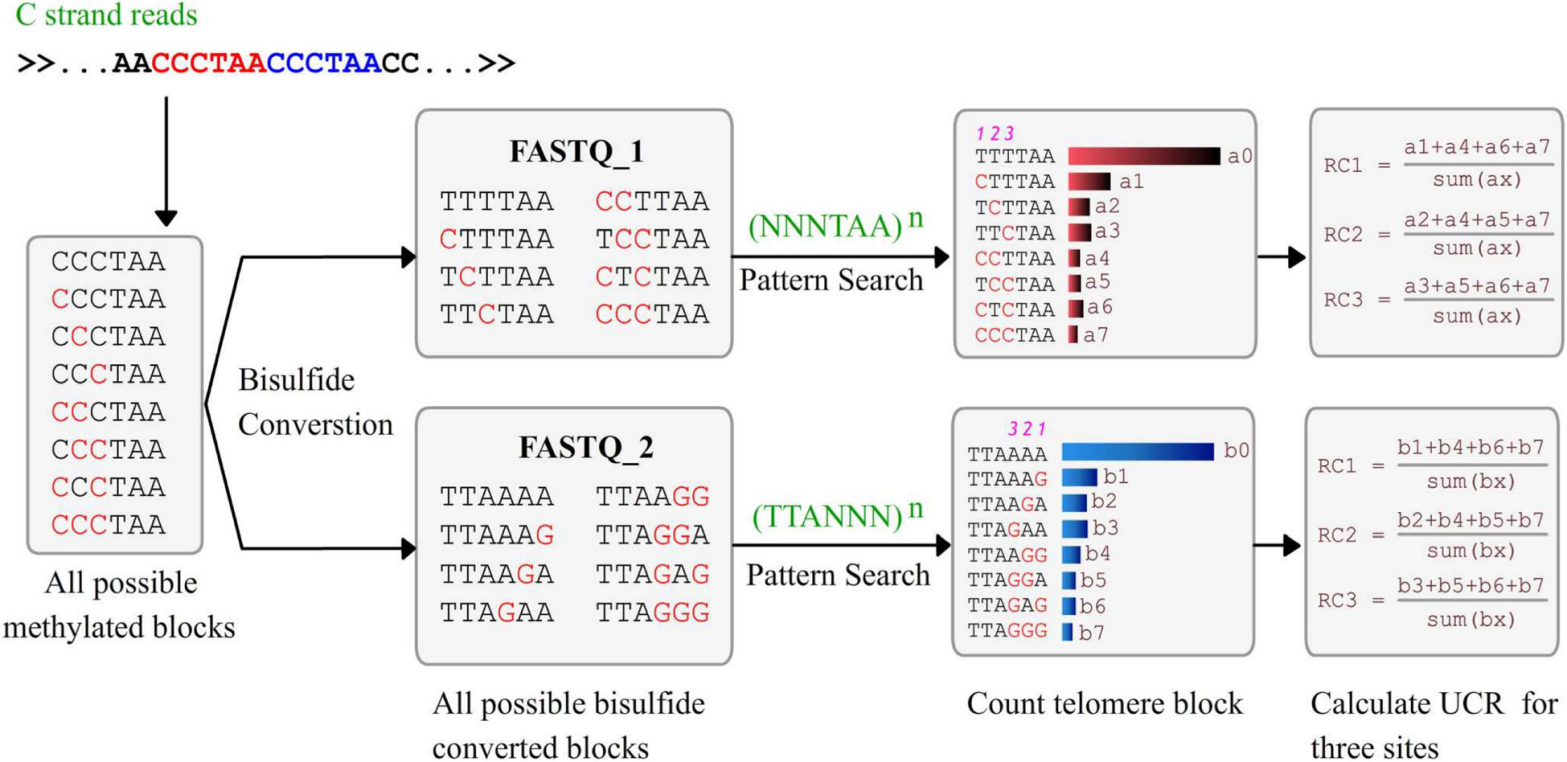
The diagram about the procedure to calculate UCRs for three non-CpG cytosine sites using C-strand original reads. In the patterns, the N represents G or C

The procedure of WGBS has been fully descripted other places [8]. Specifically, the way that telomeric DNA is processed in WGBS is illustrated in Fig. 3. It can be summarized as four steps: I. Double strand telomeric DNA fragments are composed by G-strand (5′-(TTAGGG)_n_-3′) and C-strand (5′(CCCTAA)_n_-3′); II. dsDNA are denatured and become ssDNAs; III. Bisulfite treatment converts unmethylated cytosines in the C strand into thymines; IV. The polymerase chain reaction (PCR) library construction to form dsDNA; V. Next generation pair-end sequencing and data analysis. As the patterns of c-strand origin telomeric reads in two FASTQ files are known, it is easy to calculate UCR by comparing them with theoretical bisulfite covered sequences (Fig. 3). The details of our algorithm are shown in Fig. 4. The key step is searching two regular expressions ((NNNTAA) n and (TTANNN) n against FASTQ1 and FASTQ2 files respectively to find c-strand original reads, where N is A or G and the upper n is the minimal number of repeat units. The following step is counting all possible repeats and then calculating the unconverted ratios (UCRs) for all three non-CpG cytosines (Fig. 4). As both failure of bisulfite conversion and in vivo methylation at telomere are rare and independent, so UCRs can be mimic by the sum of the false conversion ratio (FCR) and methylated ratio (MR).

1 $$ \mathrm{UCR}=\mathrm{FCR}+\mathrm{MR} $$

We classified six-bases telomeric blocks (TTAGGG) into four categories based on the number of unconverted cytosines (we named them as N0~N3 blocks). We considered the unconverted events as Bernoulli trials with certain possibility (UCR). So the percentage of N0~N3 telomeric blocks (R1~R3) should fit a binomial model and following formulas:

2 $$ C{\displaystyle \begin{array}{c}1\\ {}3\end{array}}\times UCR\times {\left(1- UCR\right)}^2=R1 $$

3 $$ C{\displaystyle \begin{array}{c}2\\ {}3\end{array}}\times {UCR}^2\times \left(1- UCR\right)=R2 $$

4 $$ C{\displaystyle \begin{array}{c}3\\ {}3\end{array}}\times {UCR}^3=R3 $$

As R1, R2 and R3 can be observed, it is easy to speculate UCR theoretically using these formulas. Actually, we only used the formula 2 to calculate UCRs because the sample size for R1 is much bigger than others.

All algorithm testing and comparisons were run at a DELL PowerEdge R730 server, with 128G RAM and two Xeon E5–2600 v3 processors with 18 cores. Bismark was downloaded from here (https://github.com/FelixKrueger/Bismark) and run following its manual with default settings. For each FSATQ file, 12 million reads are randomly extracted to save testing time.

## Supplementary information

**Additional file 1: Figure S1.** The location of N7 telomeric (TTAGGG)_7_ repeats in hg38 genome. **Table S1.** Detailed analysis results.

## Data Availability

A ready to use python script is available freely for all academic users at https://github.com/hqyone/BCR_Evaluator.

## Abbreviations

BCR: Bisulfite conversion ratio
FCR: False conversion ratio
MR: Methylated ratio
PBAT: Post-bisulfite adapter tagging
PCR: Polymerase chain reaction
QC: Quality control
RRBS: Reduced representation bisulfite sequencing
UCR: Unconverted rate
WGBS: Whole genome shotgun bisulfite sequencing
