# Supplementary material for: BCREval: a computational method to estimate the bisulfite conversion ratio in WGBS
Source: BMC Bioinformatics. 2020 Jan 31;21:38. doi: 10.1186/s12859-019-3334-z (PMC6995172; doi:10.1186/s12859-019-3334-z)
Supplement: Supplementary file 1 — Additional file 1: Figure S1. The location of N7 telomeric (TTAGGG)7 repeats in hg38 genome. Table S1. Detailed analysis results. [file 12859_2019_3334_MOESM1_ESM.docx]

Supplemental Materials

**A computational method to evaluate the bisulfite conversion ratio in WGBS**

Junhua Zhou^1^, Zefang Sun^1^, Mingqiong Zhao^1^, Feilong Wu^1^, Yucong Liu^1^, Xianghua Liu^2^, Quanze He^3^, Quanyuan He^1#^

Figure S1


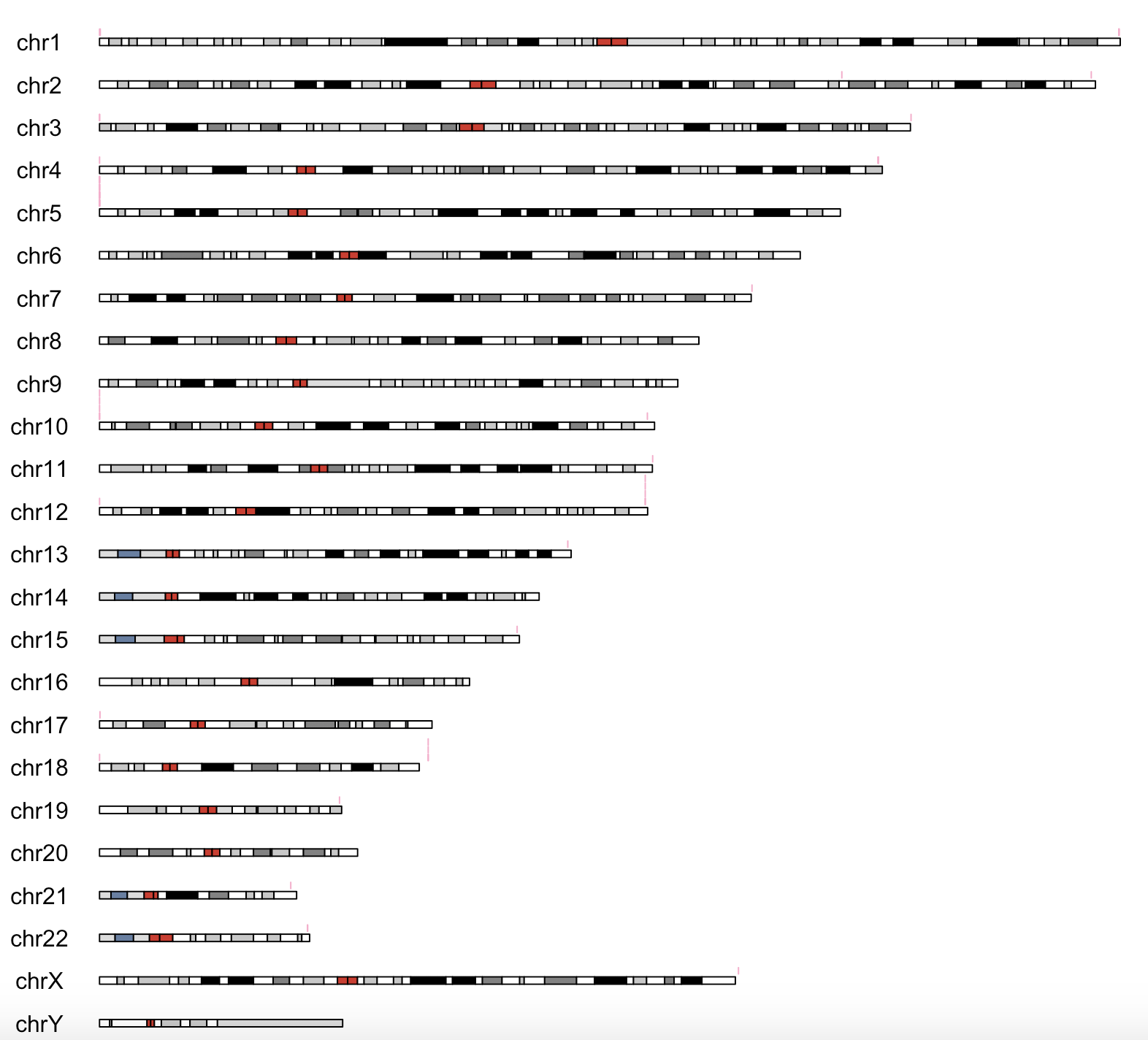


Figure S1. The location of N7 telomeric (TTAGGG)_7_ repeats in hg38 genome. This location were found by Blat at UCSC server. It is drawn by R package named karyoploteR.

Table S1


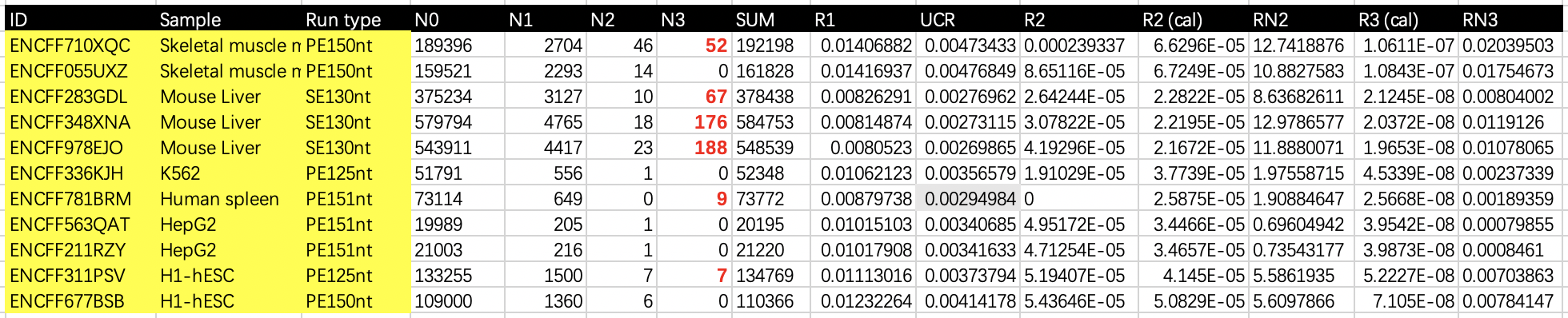


Table S1. Detailed analysis results. The titles are descripted as following: ID: the ENCODE ID of FASTQ file; Sample: short description about the sample source; Run type: The NGS sequencing type for example PE150nt indicating 150bp pair-end sequencing; N0~N3: the numbers of telomeric blocks which contain 0 to 3 unconverted cytosines respectively; SUM: the total number of telomeric blocks; R1~R3: the observed ratios of N1~N3 blocks to total; UCR: the uncovered ratio of cytosines calculated by R1. R2(cal) and R3(cal): The theoretical ratios of N2,N3 blocks to total. The calculated methods were described in text.
